# Supplementary material for: High global consumption of potentially inappropriate fixed dose combination antibiotics: Analysis of data from 75 countries
Source: PLoS One. 2021 Jan 20;16(1):e0241899. doi: 10.1371/journal.pone.0241899 (PMC7817037; doi:10.1371/journal.pone.0241899)
Supplement: S1 Table — (DOCX) [file pone.0241899.s001.docx]

**Supplementary table 1. Antibiotic FDCs in 2015 sorted by SU globally sold**

| **FDC** | **SU globally sold** | **Number of selling countries** |
| --- | --- | --- |
| amoxicillin/clavulanic acid | 8.38 x 10^9^ | 75 |
| sulfamethoxazole/trimethoprim | 3.61 x 10^9^ | 75 |
| ampicillin/cloxacillin | 0.95 x 10^9^ | 13 |
| piperacillin/tazobactam | 0.79 x 10^9^ | 66 |
| cefixime/ofloxacin | 0.31 x 10^9^ | 1 |
| metronidazole/spiramycin | 0.24 x 10^9^ | 17 |
| cefpodoxime proxetil/clavulanic acid | 0.23 x 10^9^ | 3 |
| amoxicillin/flucloxacillin | 0.19 x 10^9^ | 7 |
| azithromycin/cefixime | 0.17 x 10^9^ | 2 |
| cefoperazone/sulbactam | 0.16 x 10^9^ | 28 |
| amoxicillin/cloxacillin | 0.16 x 10^9^ | 4 |
| ampicillin/sulbactam | 0.12 x 10^9^ | 49 |
| cefixime/clavulanic acid | 0.12 x 10^9^ | 2 |
| cefalexin/trimethoprim | 0.09 x 10^9^ | 1 |
| cefuroxime axetil/clavulanic acid | 0.08 x 10^9^ | 1 |
| piperacillin/sulbactam | 0.07 x 10^9^ | 4 |
| amoxicillin/dicloxacillin | 0.07 x 10^9^ | 4 |
| mezlocillin/sulbactam | 0.06 x 10^9^ | 1 |
| cefpodoxime proxetil/ofloxacin | 0.05 x 10^9^ | 1 |
| cefixime/dicloxacillin/lactobacillus acidophilus | 0.05 x 10^9^ | 1 |
| ceftriaxone/sulbactam | 0.04 x 10^9^ | 6 |
| ceftriaxone/tazobactam | 0.04 x 10^9^ | 3 |
| azithromycin/cefpodoxime proxetil | 0.04 x 10^9^ | 1 |
| amoxicillin/cloxacillin/lactobacillus acidophilus | 0.04 x 10^9^ | 1 |
| amoxicillin/pivsulbactam | 0.04 x 10^9^ | 15 |
| amoxicillin/dicloxacillin/lactobacillus acidophilus | 0.04 x 10^9^ | 1 |
| cefixime/ornidazole | 0.03 x 10^9^ | 1 |
| cefpodoxime proxetil/levofloxacin | 0.03 x 10^9^ | 1 |
| cefixime/linezolid | 0.03 x 10^9^ | 1 |
| cefdinir/clavulanic acid | 0.03 x 10^9^ | 1 |
| amoxicillin/sulbactam | 0.03 x 10^9^ | 14 |
| azithromycin/levofloxacin | 0.02 x 10^9^ | 1 |
| cefotaxime/sulbactam | 0.02 x 10^9^ | 2 |
| cefadroxil/clavulanic acid | 0.02 x 10^9^ | 2 |
| amoxicillin/clavulanic acid/lactobacillus acidophilus | 0.02 x 10^9^ | 2 |
| ampicillin/dicloxacillin | 0.02 x 10^9^ | 5 |
| ampicillin/flucloxacillin | 0.02 x 10^9^ | 4 |
| ampicillin/cloxacillin/lactobacillus acidophilus | 0.02 x 10^9^ | 1 |
| cefoperazone/tazobactam | 0.02 x 10^9^ | 2 |
| ciprofloxacin/tinidazole | 0.02 x 10^9^ | 5 |
| cefaclor/clavulanic acid | 0.02 x 10^9^ | 1 |
| clavulanic acid/ticarcillin | 0.02 x 10^9^ | 19 |
| cefixime/cloxacillin/lactobacillus acidophilus | 0.01 x 10^9^ | 1 |
| cefixime/cloxacillin | 0.01 x 10^9^ | 1 |
| cefuroxime/clavulanic acid | 0.01 x 10^9^ | 2 |
| amoxicillin/bacillus coagulans/cloxacillin | 0.01 x 10^9^ | 1 |
| azithromycin/ofloxacin | 0.01 x 10^9^ | 1 |
| cefpodoxime proxetil/dicloxacillin/lactobacillus acidophilus | 0.01 x 10^9^ | 1 |
| cefuroxime axetil/linezolid | 0.01 x 10^9^ | 1 |
| ciprofloxacin/metronidazole | 0.01 x 10^9^ | 1 |
| cefadroxil/trimethoprim | *0.08 x 10^8^ | 1 |
| acetylspiramycin/metronidazole | 0.08 x 10^8^ | 1 |
| sulfadiazine/sulfamethoxazole/trimethoprim | 0.08 x 10^8^ | 1 |
| ampicillin/lidocaine/sulbactam | 0.05 x 10^8^ | 1 |
| ofloxacin/ornidazole | 0.04 x 10^8^ | 6 |
| ampicillin/dicloxacillin/lactobacillus acidophilus | 0.04 x 10^8^ | 1 |
| cefixime/dicloxacillin | 0.04 x 10^8^ | 1 |
| fosfomycin/trimethoprim | 0.04 x 10^8^ | 1 |
| ampicillin/oxacillin | 0.04 x 10^8^ | 2 |
| cefpodoxime proxetil/cloxacillin/lactobacillus acidophilus | 0.03 x 10^8^ | 1 |
| amoxicillin/bacillus coagulans/dicloxacillin | 0.03 x 10^8^ | 1 |
| cefpodoxime proxetil/dicloxacillin | 0.03 x 10^8^ | 1 |
| amoxicillin/metronidazole | 0.02 x 10^8^ | 3 |
| phenazopyridine/sulfamethoxazole/trimethoprim | 0.02 x 10^8^ | 1 |
| ceftazidime/tazobactam | 0.02 x 10^8^ | 2 |
| cefixime/moxifloxacin | 0.02 x 10^8^ | 1 |
| ciprofloxacin/ornidazole | 0.02 x 10^8^ | 3 |
| cefepime/tazobactam | 0.02 x 10^8^ | 1 |
| metronidazole/norfloxacin | 0.02 x 10^8^ | 1 |
| sulfadiazine/trimethoprim | 0.01 x 10^8^ | 3 |
| erythromycin/trimethoprim | 0.01 x 10^8^ | 1 |
| erythromycin/sulfamethoxazole/trimethoprim | 0.01 x 10^8^ | 1 |
| amoxicillin/cloxacillin/dl/lactic acid | 0.01 x 10^8^ | 1 |
| sulfametrole/trimethoprim | 0.01 x 10^8^ | 4 |
| amoxicillin/clavulanic acid/nimesulide | 0.01 x 10^8^ | 1 |
| cefixime/levofloxacin | *0.07 x 10^7^ | 1 |
| azithromycin/cefixime/lactobacillus acidophilus | 0.07 x 10^7^ | 1 |
| ascorbic acid/metamizole sodium/penicillin g /streptomycin | 0.06 x 10^7^ | 1 |
| azithromycin/fluconazole/secnidazole | 0.06 x 10^7^ | 3 |
| cefpodoxime proxetil/sulbactam | 0.04 x 10^7^ | 2 |
| cefixime/lactobacillus acidophilus/ofloxacin | 0.04 x 10^7^ | 1 |
| oleandomycin/tetracycline | 0.03 x 10^7^ | 1 |
| meropenem/sulbactam | 0.03 x 10^7^ | 1 |
| cefuroxime/sulbactam | 0.03 x 10^7^ | 1 |
| penicillin g/streptomycin | 0.02 x 10^7^ | 3 |
| amoxicillin/dicloxacillin/saccharomyces boulardii | 0.02 x 10^7^ | 1 |
| levofloxacin/metronidazole | 0.02 x 10^7^ | 1 |
| ceftolozane/tazobactam | 0.01 x 10^7^ | 9 |
| meropenem/sodium/sulbactam | 0.01 x 10^7^ | 1 |
| kanamycin/penicillin g | 0.01 x 10^7^ | 1 |
| rifampicin/trimethoprim | 0.01 x 10^7^ | 2 |
| levofloxacin/ornidazole | 0.01 x 10^7^ | 2 |
| sulfamethizole/trimethoprim | *0.09 x 10^6^ | 2 |
| cefixime/cefpodoxime proxetil | 0.09 x 10^6^ | 1 |
| ceftazidime/sulbactam | 0.08 x 10^6^ | 4 |
| amoxicillin/cloxacillin/lactobacillus lactis | 0.07 x 10^6^ | 1 |
| avibactam/ceftazidime | 0.07 x 10^6^ | 2 |
| bromelains/doxycycline/lactobacillus reuteri/lactobacillus rhamnosus/ornidazole | 0.06 x 10^6^ | 1 |
| chloramphenicol/tetracycline | 0.07 x 10^6^ | 1 |
| gatifloxacin/ornidazole | 0.04 x 10^6^ | 1 |
| amikacin/cefepime | 0.04 x 10^6^ | 1 |
| doxycycline/tinidazole | 0.04 x 10^6^ | 1 |
| ampicillin/cloxacillin/saccharomyces boulardii | 0.04 x 10^6^ | 1 |
| ceftriaxone/vancomycin | 0.03 x 10^6^ | 1 |
| ampicillin/bacillus coagulans/cloxacillin | 0.03 x 10^6^ | 1 |
| dalfopristin/quinupristin | 0.02 x 10^6^ | 3 |
| amoxicillin/clavulanic acid/lactic ferments | 0.01 x 10^6^ | 1 |
| cefepime/sulbactam | 0.01 x 10^6^ | 1 |
| sulfamoxole/trimethoprim | 0.01 x 10^6^ | 1 |
| cefixime/clavulanic acid/lactobacillus acidophilus | *0.08 x 10^5^ | 1 |
| bromhexine/sulfamethoxazole/trimethoprim | 0.07 x 10^5^ | 1 |
| amoxicillin/flucloxacillin/lactobacillus acidophilus | 0.06 x 10^5^ | 1 |
| ceftazidime/tobramicin | 0.05 x 10^5^ | 1 |
| cefuroxime axetil/sulbactam | 0.04 x 10^5^ | 1 |
| amoxicillin/cloxacillin/serrapeptase | 0.01 x 10^5^ | 1 |
| ampicillin/sultamicillin | 0.01 x 10^5^ | 1 |
| ceftibuten/clavulanic acid | *0.09 x 10^4^ | 1 |
| amoxicillin/cloxacillin/lactobacillus acidophilus/serrapeptase | 0.01 x 10^4^ | 1 |
| metronidazole/tetracycline | 0.01 x 10^4^ | 1 |

*Different power
