# Supplementary material for: High global consumption of potentially inappropriate fixed dose combination antibiotics: Analysis of data from 75 countries
Source: PLoS One. 2021 Jan 20;16(1):e0241899. doi: 10.1371/journal.pone.0241899 (PMC7817037; doi:10.1371/journal.pone.0241899)
Supplement: S2 Table — (DOCX) [file pone.0241899.s002.docx]

**Supplementary table 2. Antibiotic FDC categories not approved by FDA**

| **Not FDA approved FDC categories** | **FDCs** | **SU** |
| --- | --- | --- |
| Aminopenicillin / β-lactamase resistant penicillin +/- other agents | 19 | 1.54 x 10^9^ |
| 3^rd^-4-5^th^ gen. cephalosporins / β-lactamase inhibitor +/- other agents | 13 | 0.55 x 10^9^ |
| Cephalosporins / Fluoroquinolones | 6 | 0.40 x 10^9^ |
| 1^st^-2^nd^ gen. cephalosporins / β-lactamase inhibitor +/- other agents | 8 | 0.26 x 10^9^ |
| Macrolide/ 5-nitroimidazole | 3 | 0.25 x 10^9^ |
| Macrolide/cephalosporin +/- other agents | 3 | 0.21 x 10^9^ |
| Antipseudomonal penicillin /β-lactamase inhibitor | 2 | 0.14 x 10^9^ |
| Cephalosporin/ β-lactamase resistant penicillin +/- other agents | 7 | 0.10 x 10^9^ |
| Cephalosporin/trimethoprim | 2 | 0.10 x 10^9^ |
| Aminopenicillin /β-lactamase inhibitor +/- other agents | 8 | 0.09 x 10^9^ |
| Cephalosporin/oxazolidinone | 2 | 0.04 x 10^9^ |
| Fluoroquinolone/ 5-nitroimidazole | 8 | 0.04 x 10^9^ |
| Macrolide / fluoroquinolone +/- other agents | 2 | 0.04 x 10^9^ |
| Cephalosporin/5-nitroimidazole | 1 | 0.03 x 10^9^ |
| Sulphonamides/trimethoprim +/- other agents | 9 | 0.01 x 10^9^ |
| Other combinations | 19 | 0.01 x 10^9^ |
