# Supplementary material for: High global consumption of potentially inappropriate fixed dose combination antibiotics: Analysis of data from 75 countries
Source: PLoS One. 2021 Jan 20;16(1):e0241899. doi: 10.1371/journal.pone.0241899 (PMC7817037; doi:10.1371/journal.pone.0241899)
Supplement: S3 Table — (DOCX) [file pone.0241899.s003.docx]

**Supplementary table 3. Total antibiotic sales and FDCs not approved by the US FDA as a percentage of total sales and of total antibiotic FDC sales per each country selling at least one antibiotic FDC not approved by US FDA, sorted by the highest not approved FDCs/ antibiotics.**

| **Country** | **Antibiotic sales (Standard Unite)** | **FDCs not approved by US FDA / Total antibiotic sales (%)** | **FDCs not approved by US FDA / Total antibiotic FDC sales (%)** |
| --- | --- | --- | --- |
| India | 14.74 x 10^9^ | 16.6% | 50.4% |
| Egypt | 1.72 x 10^9^ | 13.0% | 27.0% |
| Lebanon | 0.09 x 10^9^ | 8.0% | 20.1% |
| Pakistan | 3.55 x 10^9^ | 5.8% | 19.3% |
| Honk Kong | 0.12 x 10^9^ | 5.6% | 18.5% |
| Tunisia | 0.35 x 10^9^ | 4.8% | 19.7% |
| Morocco | 0.36 x 10^9^ | 4.7% | 13.7% |
| Vietnam | 1.69 x 10^9^ | 4.7% | 26.8% |
| France | 1.45 x 10^9^ | 3.7% | 13.5% |
| Bangladesh | 1.53 x 10^9^ | 3.6% | 46.0% |
| China | 13.19 x 10^9^ | 3.4% | 39.8% |
| Jordan | 0.09 x 10^9^ | 3.3% | 9.7% |
| Bulgaria | 0.10 x 10^9^ | 3.3% | 12.1% |
| Korea | 1.39 x 10^9^ | 3.2% | 11.5% |
| Turkey | 2.05 x 10^9^ | 3.0% | 7.1% |
| UAE | 0.17 x 10^9^ | 2.9% | 8.8% |
| Uruguay | 0.04 x 10^9^ | 2.8% | 12.2% |
| Spain | 1.04 x 10^9^ | 2.7% | 7.4% |
| Fr. West Africa | 0.69 x 10^9^ | 2.1% | 7.6% |
| Algeria | 1.02 x 10^9^ | 1.8% | 6.5% |
| Ecuador | 0.20 x 10^9^ | 1.6% | 5.9% |
| Taiwan | 0.50 x 10^9^ | 1.2% | 11.5% |
| Russia | 1.67 x 10^9^ | 1.0% | 5.6% |
| Ukraine | 0.67 x 10^9^ | 1.0% | 11.8% |
| S. Africa | 1.16 x 10^9^ | 0.9% | 2.5% |
| Finland | 0.15 x 10^9^ | 0.9% | 18.4% |
| Mexico | 0.51 x 10^9^ | 0.8% | 3.5% |
| Austria | 0.09 x 10^9^ | 0.8% | 2.6% |
| Argentina | 0.44 x 10^9^ | 0.6% | 2.9% |
| Kazakhstan | 0.50 x 10^9^ | 0.6% | 5.1% |
| Japan | 2.22 x 10^9^ | 0.4% | 6.4% |
| C. America | 0.15 x 10^9^ | 0.3% | 1.6% |
| Kuwait | 0.02 x 10^9^ | 0.3% | 0.9% |
| Dominican Rep | 0.04 x 10^9^ | 0.3% | 2.9% |
| Brazil | 1.48 x 10^9^ | 0.2% | 1.1% |
| Latvia | 0.02 x 10^9^ | 0.1% | 0.5% |
| Greece | 0.23 x 10^9^ | 0.1% | 0.5% |
| Peru | 0.27 x 10^9^ | 0.1% | 1.4% |
| UK | 1.39 x 10^9^ | 0.1% | 1.1% |
| Colombia | 0.34 x 10^9^ | 0.1% | 1.2% |
| Romania | 0.47 x 10^9^ | 0.1% | 0.3% |
| Malaysia | 0.39 x 10^9^ | 0.1% | 0.7% |
| Slovakia | 0.08 x 10^9^ | 0.1% | 0.3% |
| Indonesia | 2.03 x 10^9^ | 0.1% | 0.9% |
| Germany | 0.80 x 10^9^ | 0.0% | 0.3% |
| Czech Republic | 0.12 x 10^9^ | 0.0% | 0.1% |
| Lithuania | 0.03 x 10^9^ | 0.0% | 0.1% |
| Poland | 0.48 x 10^9^ | 0.0% | 0.0% |
| Venezuela | 0.42 x 10^9^ | 0.0% | 0.0% |
| Thailand | 1.17 x 10^9^ | 0.0% | 0.0% |
| Philippines | 0.55 x 10^9^ | 0.0% | 0.0% |
| Sri Lanka | 0.21 x 10^9^ | 0.0% | 0.0% |
| Saudi Arabia | 0.60 x 10^9^ | 0.0% | 0.0% |
