# Supplementary material for: High global consumption of potentially inappropriate fixed dose combination antibiotics: Analysis of data from 75 countries
Source: PLoS One. 2021 Jan 20;16(1):e0241899. doi: 10.1371/journal.pone.0241899 (PMC7817037; doi:10.1371/journal.pone.0241899)
Supplement: S4 Table — (DOCX) [file pone.0241899.s004.docx]

**Supplementary table 4. Antibiotic FDCs compatible with WHO EML and AWaRe classes**

| **FDCs 2015** | **SU globally sold 2015** | **2017 WHO EML** |
| --- | --- | --- |
| amoxicillin/clavulanic acid | 8.38 x 10^9^ | Access |
| sulfamethoxazole/trimethoprim | 3.61x 10^9^ | Access |
| piperacillin/tazobactam | 0.79 x 10^9^ | Watch |
| cefpodoxime proxetil/clavulanic acid | 0.23 x 10^9^ | Watch |
| cefoperazone/sulbactam | 0.16 x 10^9^ | Watch |
| cefixime/clavulanic acid | 0.12 x 10^9^ | Watch |
| mezlocillin/sulbactam | 0.06 x 10^9^ | Watch |
| ceftriaxone/sulbactam | 0.04 x 10^9^ | Watch |
| ceftriaxone/tazobactam | 0.04 x 10^9^ | Watch |
| cefdinir/clavulanic acid | 0.03 x 10^9^ | Watch |
| cefotaxime/sulbactam | 0.02 x 10^9^ | Watch |
| cefoperazone/tazobactam | 0.02 x 10^9^ | Watch |
| clavulanic acid/ticarcillin | 0.02 x 10^9^ | Watch |
| ceftazidime/tazobactam | 0.02 x 10^8^ | Watch |
| cefepime/tazobactam | 0.02 x 10^8^ | Reserve |
| cefpodoxime proxetil/sulbactam | 0.04 x 10^7^ | Watch |
| ceftolozane/tazobactam | 0.01 x 10^7^ | Reserve |
| ceftazidime/sulbactam | 0.07 x 10^6^ | Watch |
| avibactam/ceftazidime | 0.07 x 10^6^ | Watch |
| cefepime/sulbactam | 0.01 x 10^6^ | Reserve |
| ceftibuten/clavulanic acid | 0.09 x 10^5^ | Watch |
