# Supplementary material for: High global consumption of potentially inappropriate fixed dose combination antibiotics: Analysis of data from 75 countries
Source: PLoS One. 2021 Jan 20;16(1):e0241899. doi: 10.1371/journal.pone.0241899 (PMC7817037; doi:10.1371/journal.pone.0241899)
Supplement: S5 Table — (DOCX) [file pone.0241899.s005.docx]

**Supplementary table 5. Antibiotic FDCs combinations including two highest critically important antibiotics**

| **FDC** | **SU sold in 2015** | **US FDA approval** | **EML listed** | **Countries** |
| --- | --- | --- | --- | --- |
| cefixime/ofloxacin | 0.31 x 10^9^ | not | not | India |
| azithromycin/cefixime | 0.17 x 10^9^ | not | not | India, Indonesia |
| azithromycin/levofloxacin | 0.02 x 10^9^ | not | not | India |
| azithromycin/ofloxacin | 0.01 x 10^9^ | not | not | India |
| cefixime/moxifloxacin | 0.02 x 10^8^ | not | not | India |
| cefixime/levofloxacin | 0.07 x 10^7^ | not | not | India |
| azithromycin/cefixime/*L. acidophilus* | 0.07 x 10^7^ | not | not | India |
| cefixime//ofloxacin/ *L. acidophilus* | 0.04 x 10^7^ | not | not | India |
| cefixime/cefpodoxime proxetil | 0.09 x 10^6^ | not | not | India |
| ceftriaxone/vancomycin | 0.03 x 10^6^ | not | not | India |
